# Six-Year Immunologic Recovery and Virological Suppression of HIV Patients on LPV/r-Based Second-Line Antiretroviral Treatment: A Multi-Center Real-World Cohort Study in China

**DOI:** 10.3389/fphar.2019.01455

**Published:** 2019-12-11

**Authors:** Xiaojie Huang, Liumei Xu, Lijun Sun, Guiju Gao, Weiping Cai, Yanfen Liu, Haibo Ding, Hongxia Wei, Ping Ma, Min Wang, Shuiqing Liu, Yaokai Chen, Xiaohong Chen, Qingxia Zhao, Jianhua Yu, Yuxia Song, Hui Chen, Hao Wu, Shanfang Qin, Linghua Li

**Affiliations:** ^1^Center for Infectious Diseases, Beijing Youan Hospital, Capital Medical University, Beijing, China; ^2^Department of Clinical AIDS Research, the Third People’s Hospital of Shenzhen, Shenzhen, China; ^3^Clinical and Research Center for Infectious Diseases, Beijing Ditan Hospital, Capital Medical University, Beijing, China; ^4^InInfectious Diseases Center, Guangzhou Eighth People’s Hospital, Guangzhou Medical University, Guangzhou, China; ^5^Center for Infectious Diseases, the Fourth People's Hospital of Nanning, Nanning, China; ^6^NHC Key Laboratory of AIDS Immunology (China Medical University), Department of Laboratory Medicine, The First Affiliated Hospital of China Medical University, Shenyang, China; ^7^Department of Infectious Disease, The Second Hospital of Nanjing, Affiliated Nanjing Hospital of Nanjing University of Chinese Medicine, Nanjing, China; ^8^Department of Infectious Disease, the Affiliated Second Peoples’ Hospital of the Nankai University, Tianjin, China; ^9^Institute of HIV/ AIDS, The First Hospital of Changsha, Changsha, China; ^10^Department of Infectious Diseases, Guiyang Public Health Clinical Center, Guiyang, China; ^11^Division of Infectious Diseases, Chongqing Public Health Medical Center, Chongqing, China; ^12^Department of Infectious Diseases, the Fourth Affiliated Hospital of Harbin Medical University, Harbin, China; ^13^Department of Infectious Diseases, Henan Infectious Disease Hospital, Zhengzhou, China; ^14^Department of Infectious Diseases, XIXI Hospital of Hangzhou, Hangzhou, China; ^15^Department of Infectious Diseases, Xinjiang Uygur Autonomous Region Sixth People’s Hospital, Xinjiang, China; ^16^School of Biomedical Engineering, Capital Medical University, Beijing, China; ^17^Department of Infectious Diseases, Longtan Hospital of Guangxi Zhuang Autonomous Region, Liuzhou, China

**Keywords:** efficacy and safety, ART-experienced, second-line antiretroviral therapy, human immunodeficiency virus, lopinavir/ritonavir

## Abstract

The World Health Organization guidelines recommend lopinavir/ritonavir (LPV/r) as a second-line antiretroviral therapy (ART) for HIV-infected adults in middle-income and low-income countries as a protease inhibitor boost based on clinical trials; however, the real-world safety and efficacy remain unknown. Therefore, we conducted a large-scale, multicenter retrospective cohort study to evaluate the efficacy and safety of LPV/r-based ART among HIV-infected adults in China in whom first-line therapy failed. The data were obtained from a national database covering 17 clinics in China for six years of follow-up from 2009 to 2016. Failure of first-line treatment was determined according to a viral load at least 400 copies/ml at week 48, non-completers at week 48 for any reason, and those who switched ART before week 48 for any reason such as side effects. Treatment effectiveness was assessed by the rate of CD4^+^T cell recovery, defined as >500 cells/mm^3^, and the proportion of patients achieving viral suppression, defined as <400 or <50 copies/ml according to the methods used during treatment. Safety was assessed by rates of LPV/r-related adverse events (AEs), including lipid disorder, severe abnormal liver function, myelosuppression, and renal function. Between 2009 and 2016, 1196 participants (median, 36 years old; IQR, 30–43 years) were ultimately enrolled. All patients had been on LPV/r-based second-line ART treatment for more than one year after failure of any first-line ART regimen. Overall CD4^+^T cell counts increased from 138 cells/mm^3^ to 475 cells/mm^3^ and 37.2% of all participants reached CD4 recovery. Viral suppression rates dramatically increased at the end of the first year (<400 copies/ml, 88.8%; <50 copies/ml, 76.7%) and gradually increased during follow-up (<400 copies/ml, 95.8%; <50 copies/ml, 94.4%). The most frequently reported AEs were LPV/r-induced lipid disorders with no obvious increase on LDL-C at follow-up visits. This is the first real-world LPV/r-based second-line treatment study to cover such a large population in China. These results provide strong clinical evidence that LPV/r-based second-line ART is effective in increasing CD4^+^T cell counts and viral suppression rates with tolerable side effects in HIV-infected adults in China in whom first-line treatment had failed.

## Introduction

Over two thirds of new cases of HIV diagnosed globally in 2017 were estimated to have occurred in resource-limited areas, including eastern and southern Africa, western and central Africa, and Latin America (UNAIDS, 2018). Thousands of HIV-infected adults who live in these resource-limited countries nevertheless have access to antiretroviral therapy (ART) according to World Health Organization (WHO) guidelines (Gilks et al., 2006), which has largely contributed to the reduction in mortality and morbidity associated with HIV infection and has remarkably improved quality of life of people living with HIV/AIDS (PLWHA) (Mills et al., 2011; Johnson et al., 2012; Teeraananchai et al., 2017).

However, an increasing number of HIV-infected adults have shown ﬁrst-line regimen failure, requiring a switch to second-line therapy (Fox et al., 2012; Liégeois et al., 2012). Moreover, a recent study presented at the 25th Conference on Retroviruses and Opportunistic Infections (CROI 2018) demonstrated that more than half of all HIV-infected adults in low- and middle-income countries may not achieve and maintain continuous viral suppression under second-line ART. Thus, it is essential for clinicians to assess the optimum second-line ART regimen in PLWHA in resource-constrained areas in whom ﬁrst-line therapy has failed.

The WHO guidelines recommend second-line combination ART with a ritonavir-boosted protease inhibitor (PI; either lopinavir or atazanavir) combined with at least two nucleoside/nucleotide reverse transcriptase inhibitors (NRTIs). Lopinavir/ritonavir (LPV/r) is currently widely used in middle-income and low-income countries, such as China and South Africa, based on demonstrated effectiveness and safety with respect to immunological restoration and tolerable side-effects in ART-naïve and experienced patients in combination with other ART drugs in clinical trials (Cohen et al., 2005; Paton et al., 2014; Ciaffi et al., 2015; La Rosa et al., 2016). Currently, boosted PI options are recommended as part of second-line regimens because of their safety and efficacy as indicated by systematic reviews and meta-analyses (Hermes et al., 2012; Huang et al., 2018). However, there is still no solid real-world evidence for the long-term safety and efficacy of LPV/r as second-line therapy in resource-limited settings.

Many factors can potentially influence real-world efficacy (Sherman et al., 2016), including adherence, the first-line ART regimen, baseline CD4 counts, viral load, and age, before switching to second-line treatment, which could limit the external validity of traditional randomized clinical trials (RCTs). Furthermore, the present WHO guideline was based on the results of RCTs in limited countries; thus, more evidence on efficacy is required to support decisions on treatment (Wang et al., 2009; Sherman et al., 2016). As a result, there is an urgent need for more data on the long-term real-world efficacy of this widely used second-line regimen to enable clinicians to make informed judgements for patient selection in resource-constrained areas for whom first-line therapy has failed.

Therefore, we conducted the present Chinese multi-center real-world cohort study to provide suitable data on the efficacy and safety of second-line ART with LPV/r for all patients in whom ﬁrst-line ART had failed, which can help develop standard guidelines for treatment.

## Methods

### Study Design and Participants

This large-scale multi-center retrospective study was conducted using data collected from a national database from 2009 to 2016 across 17 clinics in China (Jing et al., 2017). This study was reviewed and approved by the Beijing Youan Hospital institutional board, which was the leading research institute for this study. Eligibility criteria for included participants were as follows: 1) adults 18 years or older, and 2) failure of any first-line ART regimen (viral load of least 400 copies/ml at week 48), followed by LPV/r-based second-line ART. In brief, each center collected data from existing national databases on demographics (age, gender), baseline information (CD4^+^T cell counts, viral load, and WHO stage), ART treatment history, and HIV-related diseases. There was no adverse events (AEs) information in the national databases; therefore, details on AEs were collected separately according to participants’ medical records.

### Procedures

The efficacy of the second-line LPV/r regimen was evaluated according to the immunological and virological responses at baseline and at 6, 12, and 72 months on ART.

Good immune recovery was defined as a CD4^+^T cell count more than 500 cells/mm^3^, and the percentage of viral suppression was defined as a viral load below 50 or 400 copies/ml. WHO-defined stage IV disease was determined according to the WHO clinical staging of HIV disease in adults. Factors related to recovery of CD4^+^T cell counts and WHO-defined stage IV disease were also analyzed. The safety of second-line LPV/r regimens was evaluated according to the rates of drug-related AEs (see **Table S1** for definitions of each AE). An AE was considered if any result in the follow-up visit was abnormal.

### Statistical Analysis

Baseline demographic and clinical data were stratified according to the baseline age of participants (<50 years or ≥50 years). Descriptive statistics are presented as medians with interquartile ranges (IQRs) or counts with proportions as appropriate. Two-sample *t*-tests were used to compare means, and χ^2^ tests were used to compare proportions. Fisher’s exact test was used when there were fewer than 40 participants or when expected values were lower than 1 in 20% of the cells for R × C tables. Correction for continuity was performed when there were more than 40 participants and expected values were between 1 and 5 in 20% of the cells for R × C tables. The Cox proportional-hazards model was used to investigate associations between baseline levels of CD4^+^ T cells, viral load, or age of participants and good immune reconstitution. Factors associated with baseline WHO-defined Stage IV disease were identified by logistic regression model. Risk ratios (RRs) for good immune recovery were estimated with 95% confidence intervals (CIs). All *P* values were two-sided, and *P* < 0.05 was considered statistically significant. The data were analyzed using SPSS version 24.0 for Windows (SPSS Inc., Chicago, IL).

## Results

### Participant Selection and Baseline Characteristics

Between 2009 and 2016, 4006 patients used LPV/r as a second-line drug. Among these, 2078 patients with a viral load below 400 copies/ml at baseline were excluded from the study, leaving a total of 1928 patients in the first-line treatment failure group. A further 53 patients below 18 years of age, 556 patients who were undergoing the second-line treatment for less than 1 year, and 23 duplicate records were excluded, resulting in a total 1196 participants who failed any first-line ART regimen and switched to LPV/r-based second-line ART enrolled in the study.

The median age of the eligible participants was 36 years (IQR, 30–43 years). There were fewer co-infections with hepatitis C virus among those older than 50 years than among the younger patients. Distributions of routes of transmission also significantly differed among the two age groups (*P* < 0.001). Detailed information of the other differences in baseline characteristics according to age group is shown in **Table 1**.

**Table 1:** Baseline characteristics of included participants.

	Age ≤ 50 years (n = 1034)	Age > 50 years (n = 121)	Overall^#^ (n = 1155)	*P* value
Male gender, n (%)	862 (83.4%)	100 (82.6%)	962 (83.3%)	0.841
Time since HIV diagnosis (months) median (IQR)	24 (15,39)	21 (12,39)	24 (15,39)	0.434
Route of infection				<0.001
Blood transfusion	19 (1.8%)	1 (0.8%)	20 (1.7%)	
Plasma	5 (0.5%)	1 (0.8%)	6 (0.5%)	
Drug injection	171(16.5%)	4 (3.3%)	175 (15.2%)	
Homosexual sexual transmission	340 (32.9%)	23 (19.0%)	363 (31.4%)	
Heterosexual sexual transmission	430 (41.6%)	80 (66.1%)	510 (44.2%)	
Other	69 (6.7%)	12 (9.9)	81 (7.0%)	
Co-infection with HCV, n (%)	66 (6.4%)	2 (1.7%)	68 (5.9%)	0.036
Co-infection with HBV, n (%)	66 (6.4%)	5(4.1%)	71 (6.1%)	0.329
HIV-1 RNA (log copies/ml), median (IQR)	4.4 (3.7–5.0)	4.4 (3.8–5.1)	4.4 (3.7–5.0)	0.500
HIV-1 RNA < 3	43 (4.2%)	3 (2.5%)	46 (4.0%)	0.517
HIV-1 RNA ≥ 3	991 (95.8%)	118 (97.5%)	1109 (96.0%)	
Baseline CD4+T-cell count (cells/mm^3^), median (IQR)	141 (57–265)	119 (48–225)	138 (54–262)	0.095
Baseline CD4+T-cell count < 350	891 (86.2%)	108 (89.3%)	999 (86.5%)	0.385
Baseline CD4+T-cell count ≥ 350	140 (13.5%)	13 (10.7%)	153 (13.2%)	
Missing	3 (0.3%)	0	3 (0.3%)	

^#^41 participants were missing baseline data on age.

### CD4^+^T Cell Counts Recovery

**Figure 1:**
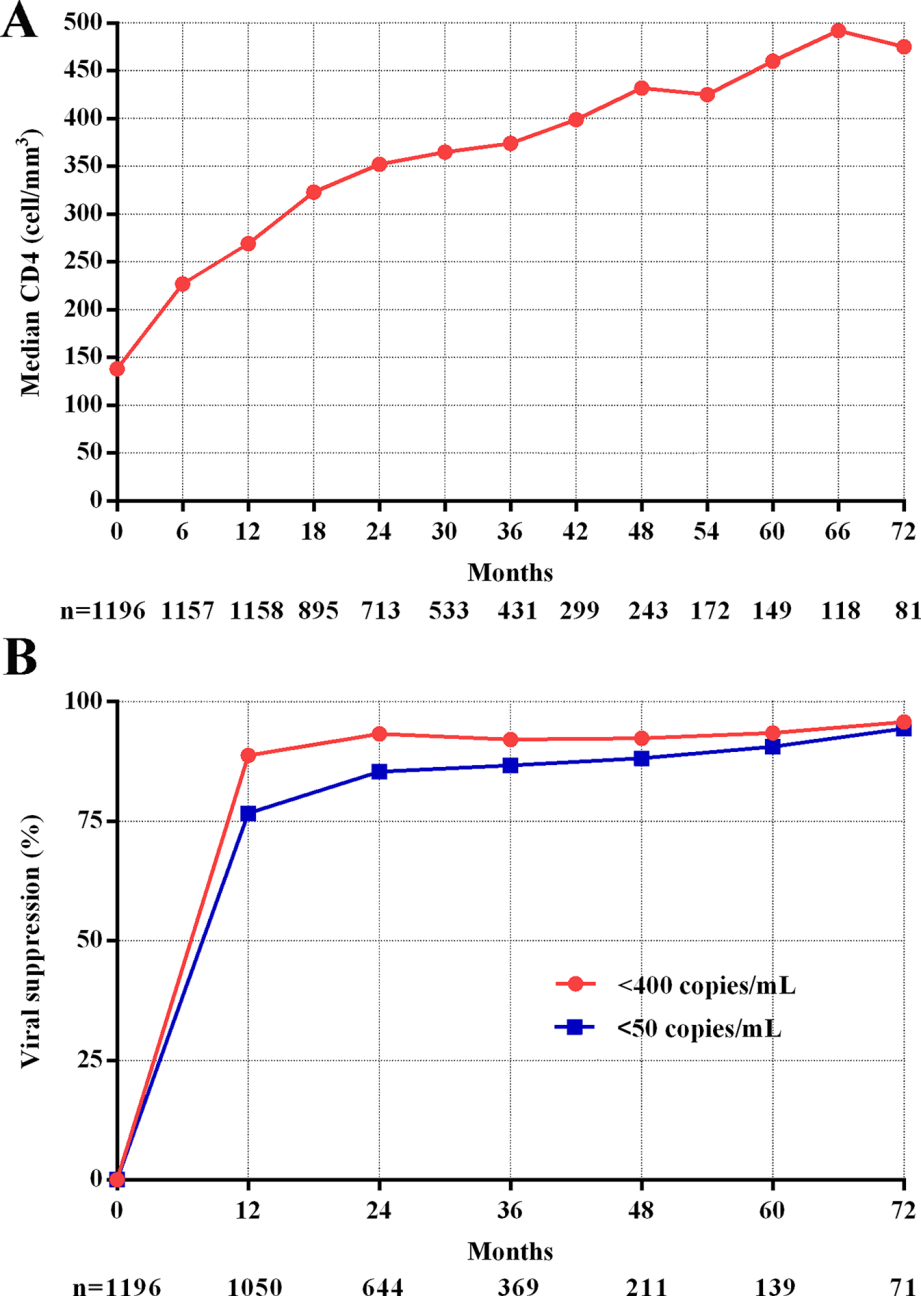
CD4 recovery and viral suppression during follow-up. **(A)** Trend of median CD4+T cell counts. **(B)** Trend of viral suppression.

**Figure 2:**
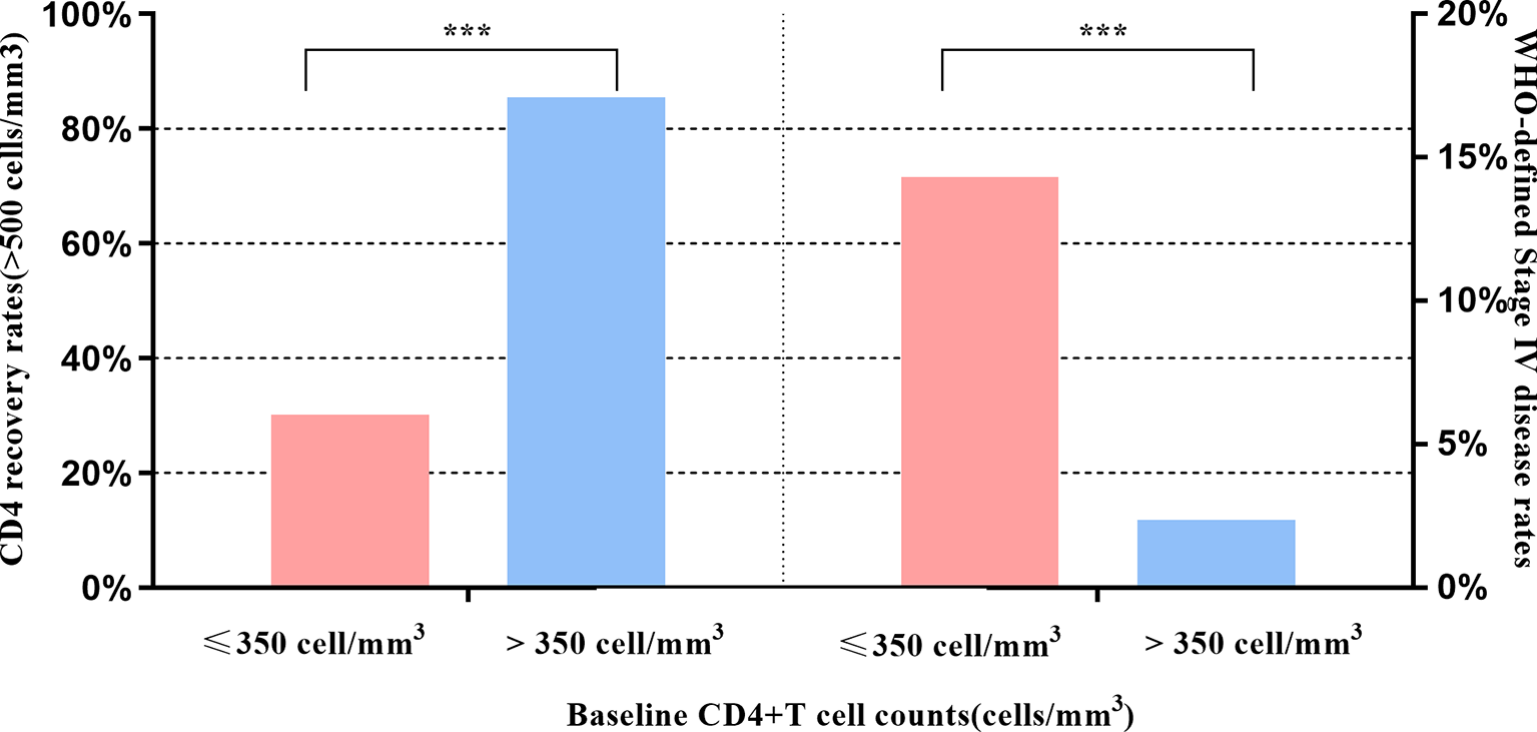
Effects of baseline CD4 on CD4 recovery and WHO-defined stage four disease. *** indicates P < 0.001.

**Figure 3:**
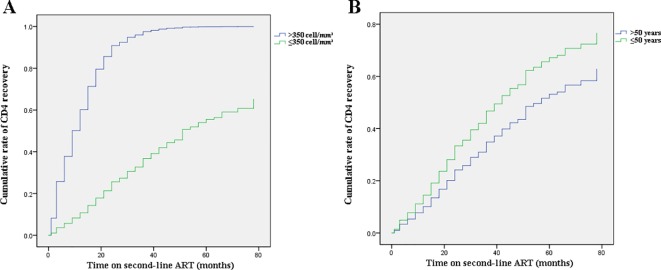
Effects of baseline CD4+T cell counts and age on change trends of CD4+T cell counts. **(A)** Baseline CD4+T cell count-stratified results on trends of CD4+T cell counts. **(B)** Age-stratified results on trends of CD4+T cell counts.

**Figure 1A** shows the dramatically increasing trend of median CD4^+^T cell counts over the six years of LPV/r treatment. Patients with baseline CD4^+^T cell counts > 350 cells/mm^3^ showed significantly higher immunological recovery and a lower WHO-defined stage IV HIV-related disease rate than those with counts below 350 cells/mm^3^ (**Figure 2**). In univariate analyses, factors significantly associated with CD4 recovery included baseline CD4^+^T cell count, baseline viral load, and age. There was no significant association with tenofovir disoproxil fumarate (TDF)-containing treatment. In the final multivariate model, factors associated with CD4 recovery included baseline CD4^+^T cell count and age (**Table 2**). The age and baseline CD4^+^T cell count-stratified results on change trends of CD4^+^T cell counts are also shown in **Figure 3**.

**Table 2:** Factors associated with CD4 recovery.

	CD4 recovery rate n (%)	Unadjusted RR (CI)	*P* value	Adjusted RR (CI)	*P* value
Baseline CD4 > 350 cells/mm^3^	136 (85.5%)	8.2 (6.62–10.17)	<0.001	8.09 (6.51–10.06)	<0.001
Baseline VL ≤ 10^5^ copies/ml	370 (41.0%)	1.57 (1.23–2.02)	<0.001		0.216
Baseline age ≤ 50 years	402 (38.9%)	1.63 (1.13–2.35)	0.009	1.47 (1.02–2.12)	0.039
TDF-containing regimen	353 (41.6%)	1.22 (0.97–1.54)	0.088		0.105

RR, relative risk; CI, confidence interval; VL, viral load; TDF, tenofovir disoproxil fumarate.

### Viral Suppression

The viral suppression rate was much higher at the end of the first year (<400 copies/ml, 88.8%, <50 copies/ml, 76.7%) and showed a slow increase during following-up (<400 copies/ml, 95.8%, <50 copies/ml, 94.4%, **Figure 1B**).

### Who-Defined Stage IV HIV-Related Disease

In both univariate and multivariate analyses, baseline CD4^+^T cell count and baseline viral load were the only significant factors associated with baseline WHO-defined stage IV disease (**Table 3**). There was no significant association with age and TDF-containing treatment.

**Table 3:** Factors associated with baseline WHO-defined stage IV disease.

	WHO-defined stage IV disease, n (%)	Unadjusted OR (CI)	*P* value	Adjusted OR (CI)	*P* value
Baseline CD4 > 350 cells/mm^3^	3 (2.4%)	7.05 (2.20–22.57)	0.001	5.25 (1.62–16.94)	0.006
Baseline VL ≤ 10^5^ copies/ml	68 (9.8%)	0.37 (0.24–0.56)	<0.001	0.42 (0.27–0.65)	<0.001
Baseline age ≤ 50 years	96 (12.2%)	0.81 (0.43–1.56)	0.536		0.623
TDF-containing regimen	79 (12.2%)	0.84 (0.55–1.29)	0.430		0.883

OR, odds ratio; CI, confidence interval; VL, viral load; TDF, tenofovir disoproxil fumarate.

### Adverse Events

**Figure 4:**
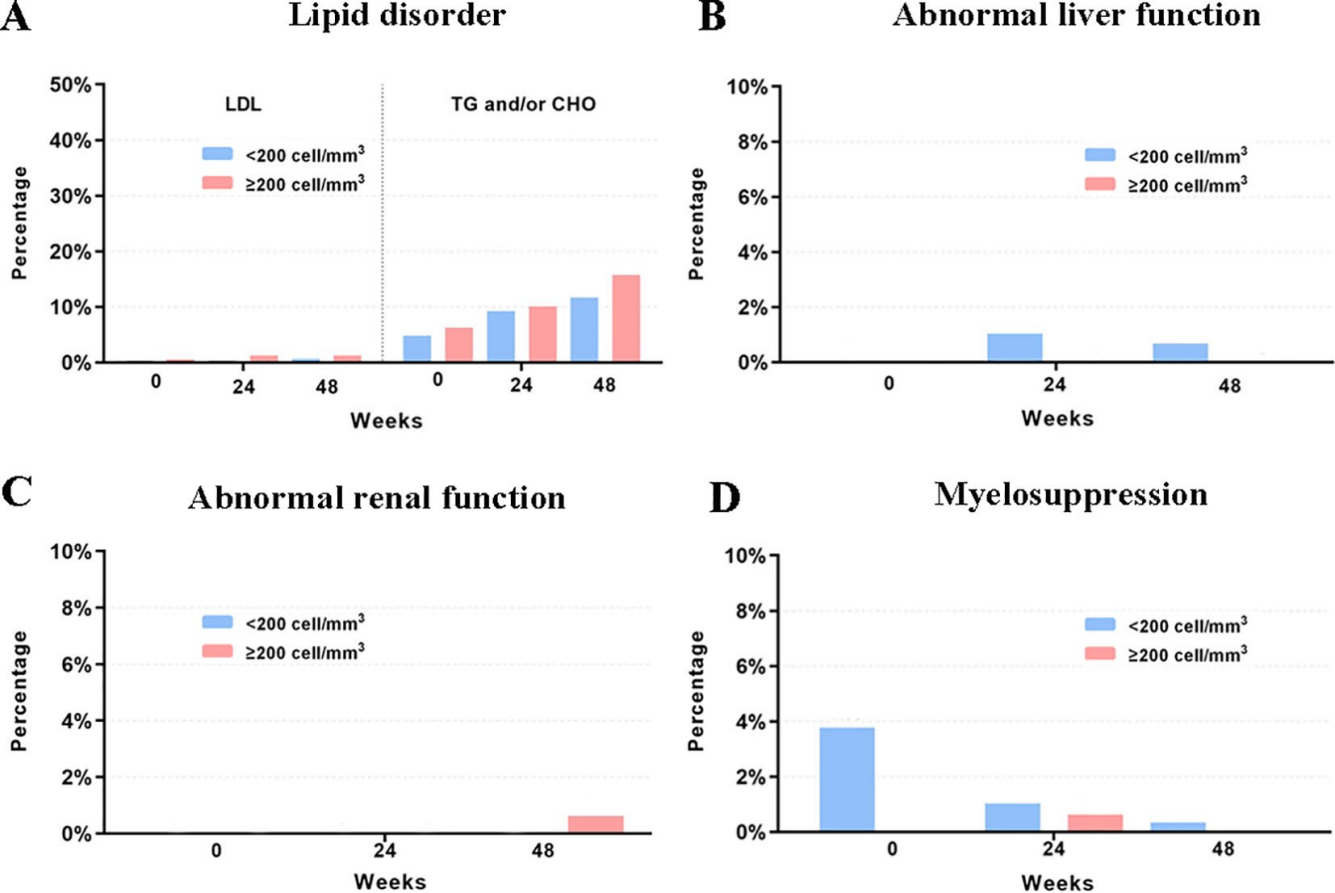
Rates of LPVr related AEs across baseline, week 24 and week 28. **(A)** The rates of grade 3 to 4 lipid disorder. **(B)** The rates of grade 3 to 4 abnormal liver function. **(C)** The rates of grade 3 to 4 abnormal renal function. **(D)** The rates of grade 3 to 4 myelosuppression. LDL, low-density lipoprotein. TG, triglycerides. CHO, cholesterol.

Laboratory data of 327, 83, 364, and 306 participants related to AEs of myelosuppression, renal function, liver function, and lipid disorder were available, respectively. Over 90% of the participants had normal myelosuppression, liver function, and renal function at baseline and during follow-up. When stratified according to the baseline CD4 count, the rates of grade 3 to 4 lipid disorder and abnormal renal function for those with ≥200 cells/mm^3^ were slightly higher than those in the patients with a baseline CD4 count <200 cell/mm^3^. Detailed information for all AEs is shown in **Table 4** and **Figure 4**.

**Table 4:** Change in severity of adverse events at baseline and follow-up.

Baseline	Follow-up^#^		*P* value
	Normal	Grade 3–4	
Myelosuppression			0.012
Normal	343 (96.6%)	1 (0.3%)	
Grade 3–4	10 (2.8%)	1 (0.3%)	
Renal function			1.000
Normal	70 (100.0%)	0	
Grade 3–4	0	0	
Liver function			1.000
Normal	133 (99.3%)	1 (0.7%)	
Grade 3–4	0	0	
Blood lipid			<0.001
Normal	24 (42.9%)	16 (28.6%)	
Grade 3–4	0	16 (28.6%)	

^#^An adverse effect was considered if any one of the follow-up visit tests was abnormal.

## Discussion

This study provides the first multicenter real-world evidence on the efficacy and safety of LPV/r-based second-line treatment in HIV patients across China, demonstrating that LPV/r-based ART is effective in increasing CD4^+^T cell counts and suppressing the viral load for patients in whom first-line treatment failed. Moreover, the patients using this second-line treatment experienced minimal drug-related AEs in this real-world setting. Age and baseline CD4^+^T cell counts were associated with CD4 recovery, and both the baseline CD4^+^T cell count and viral load were associated with viral suppression. These findings indicate that patients should be switched to second-line treatment immediately after confirmation of treatment failure.

Indeed, immunological function dramatically increased (with respect to CD4^+^T cell counts) in the first year after switching due to failure of first-line treatment, and remained stable during follow-up visits in this real-world setting, which is consistent with the results of previous observational studies and RCTs (Pujades-Rodríguez et al., 2008; Ferradini et al., 2011; Patel et al., 2013). Although not universally accepted, an increase of 100 cells/ml over the ﬁrst year on therapy can be considered as an indicator of the success of ﬁrst-line therapy given a baseline CD4^+^T cell count below 200 cells/mm^3^ (Fox et al., 2010). Thus, the average gain of 131 cells in 1 year on second-line therapy observed in the present study represents substantial immune recovery. In addition, we found that younger age and higher baseline CD4^+^T cell counts favor immune recovery owing to the preservation of thymic function (Douek et al., 1998; Viard et al., 2001). With respect to the viral load, around 95% of patients under the second-line treatment regimen achieved successful viral load suppression during the six years of follow-up, which is not inferior to the efficacy of other PIs, such as DRV/r and ATV/r (Bánhegyi et al., 2012; Akanmu et al., 2015). A higher baseline CD4^+^T cell count and a lower baseline viral load are protective factors for baseline HIV-related diseases. However, in resource-limited settings where ART failure is determined predominantly by clinical failure and immunological failure because viral load tests are not widely available and NRTIs resistance is very common (Hosseinipour et al., 2009). In the present cohort, HIV-1 RNA levels were routinely monitored (once per year) according to Chinese free ART guidelines, and thus the switch to the second-line regimen was likely made earlier than it would have been based only on clinical assessment.

Regarding the safety and tolerability of LPV/r-containing second-line regimens, we detected minimal LPV/r-related AEs in this real-world setting during the six-year follow-up period, which was consistent with previous cohort studies (Dlamini et al., 2011; Han et al., 2015). Some observational studies showed that LPV/r might be associated with the development of renal impairment, although the incidence ratios were relatively low (1.08–1.22 per year) (Mocroft et al., 2010; Ryom et al., 2013); however, we found no obvious difference in renal function compared to baseline levels. Our results highlighted that the potential for lipid disorder during LPV/r-based treatment should be carefully monitored and evaluated. Compared to LPV/r, other PIs such as DRV/r may result in a more favorable gastrointestinal and lipid profile at week 96 in spite of the non-significant discontinuation due to AEs (Mills et al., 2009). However, it is noteworthy that no obvious increase on LDL-C was found in our study, which is more relevant to the development of the atherosclerotic cardiovascular disease. In the present study, the majority of participants who had lipid disorders at baseline still had them during follow-up, which indicates that lipid profile monitoring should be integrated into standard care for patients under LPV-containing regimens.

A strength of our study is that it was performed across diverse sites in low- to middle-income areas in China with little access to other PIs, and using a real-world study design. Thus, these results can be generalizable to settings where the majority of people with HIV reside. One weakness of this study is that the retrospective design and missing data for some baseline characteristics and outcomes could have contributed to bias, which calls for caution when interpreting causal relationships for some analyses. In addition, the definition of AE as any abnormal finding during follow-up visit tests might have resulted in an overestimation of the AE prevalence. However, the use of large real-world samples from different provinces across China could improve the representativeness including participants with different demographic backgrounds, along with the higher statistical power to detect potentially significant effects.

## Conclusion

In summary, this national multicenter study contributes clear and generalizable findings to real-world settings and provides solid evidence of the suitability of LPV/r as second-line ART in resource-limited countries. Our data support the current WHO recommendation for a boosted PI plus NRTIs as second-line HIV therapy after failure of non-NRTI-based regimens in resource-limited settings.

## Data Availability Statement

Raw data is available upon request to the first author. Requests to access the datasets should be directed to Xiaojie Huang, huangxiaojie78@126.com. 

## Ethics Statement

This study was reviewed and approved by the Beijing Youan Hospital institutional board, which is the leading research institute for this study.

## Author Contributions

XH, LX, and LL led the analysis and writing of this manuscript. XH, LX, HWe, HC, HWu, SQ, and LL contributed to the final version. XH and LL designed the study. LS, GG, WC, YL, HD, PM, MW, SL, YC, XC, QZ, JY, and YS were involved in managing the data collection. All authors reviewed and approved the final version of the manuscript.

## Funding

This work was supported by the Chinese Government 13^th^ Five-Year Plan (2017ZX10201101), Major Project of Beijing Municipal Science and Technology Committee (D161100000416003, D171100000517003), the National Natural Science Foundation of China (No. 81571973), and Beijing Key Laboratory (No.BZ0089).

## Conflict of Interest

The authors declare that the research was conducted in the absence of any commercial or financial relationships that could be construed as a potential conflict of interest.
