# Supplementary material for: Six-Year Immunologic Recovery and Virological Suppression of HIV Patients on LPV/r-Based Second-Line Antiretroviral Treatment: A Multi-Center Real-World Cohort Study in China
Source: Front Pharmacol. 2019 Dec 11;10:1455. doi: 10.3389/fphar.2019.01455 (PMC6917650; doi:10.3389/fphar.2019.01455)
Supplement: Supplementary file 1 [file Table_1.docx]

Table S1 The definitions of adverse events (AEs)

|  | Grade 1  (Mild) | Grade 2  (Moderate) | Grade 3  (Severe) | Grade 4  (Life-Threatening) |
| --- | --- | --- | --- | --- |
| Myelosuppression (Hemoglobin, g/L) |  |  |  |  |
| Male only | 100 to 109 | 90 to < 100 | 70 to < 90 | < 70 |
| Female only | 95 to 104 | 85 to < 95 | 65 to < 85 | < 65 |
| Renal function (Glomerular filtration rate, ml/min) | NA | 60 to < 90 | 30 to < 60 | < 30 |
| Liver function | | | | |
| Alanine aminotransferase | 1 to < 2.5 x ULN | 2.5 to < 5 x ULN | 5 to < 10 x ULN | ≥ 10 x ULN |
| Aspartate aminotransferase | 1 to < 2.5 x ULN | 2.5 to < 5 x ULN | 5 to < 10 x ULN | ≥ 10 x ULN |
| Total bilirubin | 1.1 to < 1.6 x ULN | 1.6 to < 2.6 x ULN | 2.6 to < 5.0 x ULN | ≥ 5.0 x ULN |
| Lipid Disorders | | | | |
| Cholesterol, mmol/L | 5.18 to < 6.19 | 6.19 to < 7.77 | ≥ 7.77 | NA |
| Triglycerides, mmol/L | 1.71 to 3.42 | > 3.42 to 5.70 | > 5.70 to 11.40 | > 11.40 |
| low‐density lipoprotein, mmol/L | 3.37 to < 4.12 | 4.12 to < 4.90 | ≥ 4.90 | NA |

ULN: Upper limit of normal
